# Through the lens of schizophrenia: Recognizing negative facial expressions and family patterns

**DOI:** 10.1016/j.scog.2024.100336

**Published:** 2024-11-05

**Authors:** Leila Shateri, Hamid Yari Renani, Abbas Bakhshipour Rudsari, Touraj Hashemi Nosratabad, Zahra Saeidi

**Affiliations:** a Department of Psychiatry, Faculty of Medicine, Tabriz Medical Science University, Tabriz, Iran; b Department of Psychology, Yasuj University, Yasuj, Iran; c Department of Psychology, Faculty of Psychology & Educational Sciences, University of Tabriz, Tabriz, Iran; d Shahid Beheshti University of Medical Science, Tehran, Iran

**Keywords:** Schizophrenia, Facial emotion recognition, Paranoid vs. non-paranoid schizophrenia, Family studies, Social cognition

## Abstract

Schizophrenia is a complex disorder with symptoms such as hallucinations, delusions, and impaired social interactions, and deficits in facial emotion recognition are a key area of impairment. Studies indicate that recognizing facial emotions is essential for social interaction, and individuals with schizophrenia show significant difficulties, especially in recognizing negative emotions. Previous research has primarily focused on patients, with less attention on their first-degree relatives. This study investigates the ability to recognize negative facial expressions in paranoid and non-paranoid schizophrenia patients, their siblings, and matched healthy controls. This cross-sectional study included 60 paranoid schizophrenia patients, 60 non-paranoid schizophrenia patients, 59 siblings of paranoid patients, 60 siblings of non-paranoid patients, and 30 healthy controls, recruited from outpatients at Razi Hospital in Tabriz, Iran. The mean age was 35.7 years, and 54 % of participants were female. The Ekman 60 Faces Test assessed the recognition of basic facial emotions, focusing on negative emotions. The results revealed that paranoid schizophrenia patients showed significantly lower performance in recognizing negative facial emotions (mean score: 15.7) compared to non-paranoid patients (16.4) and siblings (28.1 for paranoid siblings, 27.4 for non-paranoid siblings). In contrast, healthy controls scored highest (29.0). This study highlights the deficits in emotion recognition in schizophrenia and their potential genetic underpinnings within family lines, contributing to understanding social cognition deficits related to the disorder.

## 1 Introduction

Schizophrenia is a debilitating mental disorder affecting approximately 1 % of the global population, characterized by symptoms such as hallucinations, delusions, disorganized thinking, and significant impairments in multiple domains of functioning, including social and role functioning (Dugan et al., 2024; Mu et al., 2024; Zhao et al., 2024). These symptoms, which include hearing voices that are not present and holding false beliefs despite evidence to the contrary, disrupt an individual's perception of reality and impair their ability to engage in everyday activities and maintain social relationships (Lane et al., 2024; Zhu et al., 2024). A significant challenge for individuals with a diagnosis of schizophrenia is their difficulty in interpreting social cues, particularly facial expressions, which are critical for effective communication and social interaction (Martínez-Cao et al., 2024). This impairment can lead to misunderstandings, social isolation, and a deterioration in interpersonal relationships, exacerbating the social withdrawal often seen in these patients (Dong et al., 2024). Notably, these impairments are hypothesized to result from deficits in processing negative emotions, such as fear and anger, which are crucial for social interactions and have been linked to underlying neurological and cognitive abnormalities specific to schizophrenia (Kohler et al., 2010). Such deficits may vary across different subgroups of schizophrenia patients, highlighting the need to examine these groups separately (Fusar-Poli et al., 2022). Investigating differences in facial emotion recognition between paranoid and non-paranoid subtypes is clinically relevant because these subtypes may exhibit distinct cognitive and emotional processing profiles, which could impact their social functioning differently (Fusar-Poli et al., 2022). Understanding these differences is crucial for tailoring interventions that address the specific needs of each group. Understanding the mechanisms contributing to these deficits is crucial for developing effective interventions to improve the social functioning of individuals with schizophrenia.

Facial emotion recognition, a key aspect of social cognition, is essential for navigating social interactions as it allows individuals to understand others' emotional states and respond appropriately (Faghel-Soubeyrand et al., 2020). Deficiencies in this ability are closely tied to the neurological aspects of schizophrenia and represent a prevalent cognitive impairment in this population, which can lead to poorer social functioning and quality of life (Gennarelli et al., 2022; Kohler et al., 2010; Pavlova et al., 2023). Previous studies have revealed that these deficits are associated with structural and functional abnormalities in brain regions such as the amygdala, which is involved in processing emotions (Allott et al., 2015; Eack et al., 2010; Montag et al., 2007). Several studies suggest that recognizing negative emotions, such as fear, anger, and sadness, poses an even greater challenge and has more significant consequences for social functioning (Eack et al., 2010; Kohler et al., 2010; Montag et al., 2007). These impairments are thought to be related to symptom formation theories, suggesting that difficulties in recognizing negative emotions may exacerbate psychotic symptoms and contribute to social dysfunction (Eack et al., 2010; Fett et al., 2013).

Endophenotypes, or measurable traits associated with schizophrenia that have a more direct genetic link, have been proposed as a way to bridge the gap between genetic variations and clinical manifestations of the disorder (Allott et al., 2015; Flint and Munafò, 2007; Gottesman and Gould, 2003; Penadés et al., 2020). Social cognition deficits have been identified as one such endophenotype, with first-degree relatives of schizophrenia patients showing impairments that, while not as severe as those of the patients themselves, still surpass those of healthy controls (Chiang et al., 2023; Eack et al., 2010; Lavoie et al., 2013; O'Reilly et al., 2015). These findings highlight the potential heritable and familial factors contributing to social cognition impairments in schizophrenia (Allott et al., 2015; Fusar-Poli et al., 2022; Lavoie et al., 2013).

Despite the extensive research on facial emotion recognition in schizophrenia, there is a lack of studies that compare different subgroups within the schizophrenia spectrum, such as paranoid and non-paranoid patients, as well as their siblings and healthy controls. Paranoid and non-paranoid subtypes are known to differ in terms of symptom severity and cognitive impairments, which could affect emotion recognition differently (Fett et al., 2013). This research aims to address this gap by exploring the differential performance of these groups on the recognition of negative emotions, which are particularly challenging for individuals with schizophrenia to recognize and have significant implications for their social functioning (Ambekar, 2023; Fusar-Poli et al., 2022; Gao et al., 2021; Montag et al., 2007). The inclusion of both paranoid and non-paranoid siblings in the study design is intended to explore the potential genetic and familial factors that may contribute to these deficits, offering new insights into the underlying mechanisms of social cognition in schizophrenia. This novel approach not only contributes to the understanding of trait-related cognitive abilities in schizophrenia but also paves the way for future research exploring the heritable aspects of social cognition in this population.

## 2 Material and methods

### 2.1 Research design

This study employed a cross-sectional comparative design to assess the ability to recognize negative facial expressions among different groups: paranoid schizophrenic patients, non-paranoid schizophrenic patients, their siblings, and healthy controls.

The cross-sectional comparative design is suitable for this study because it allows for the assessment of differences in facial emotion recognition abilities across multiple groups at a single point in time (Rajesh et al., 2021).

The philosophical underpinning of this cross-sectional comparative design is rooted in positivism, which emphasizes empirical observation and measurement to understand phenomena (Renani et al., 2024). By comparing different groups, the study seeks to identify and quantify differences in facial emotion recognition abilities objectively. This empirical approach aligns with the positivist paradigm, which values objective data collection and analysis to conclude the underlying mechanisms of social cognition deficits in schizophrenia.

### 2.2 Conceptual model

**Fig. 1:**
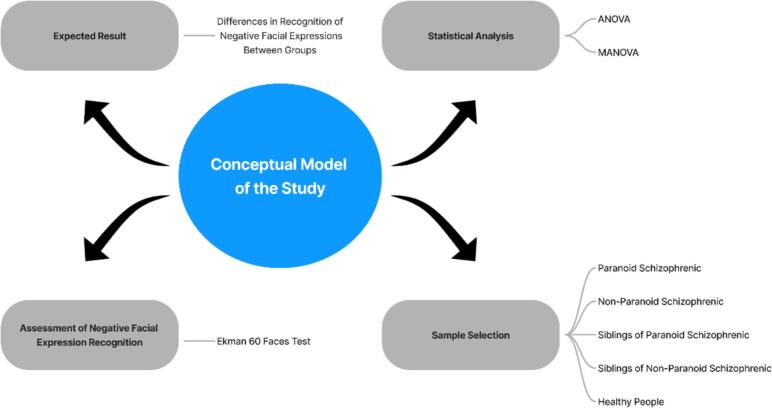
Illustrates the process of selecting participants.

Figure illustrates the conceptual model of the study, presenting the different participant groups involved in the experiment. The model includes four groups: paranoid schizophrenia patients, non-paranoid schizophrenia patients, their siblings (categorized based on the diagnosis of their sibling), and healthy controls. The primary focus is on the assessment of the recognition of negative emotions—fear, sadness, anger, and disgust—across these groups. The statistical method used is Multivariate Analysis of Variance (MANOVA) to identify differences in emotion recognition between the groups, followed by one-way Analysis of Variance (ANOVA) tests to pinpoint specific differences for each emotion. The model hypothesizes that paranoid schizophrenia patients will experience the greatest difficulty in recognizing negative emotions, followed by non-paranoid patients and their siblings, while healthy controls are expected to perform the best. The conceptual model in Fig. 1 provides an organized overview of the sample groups, the variables studied, and the statistical methods applied, guiding the analysis and interpretation of the results.

### 2.3 Sampling method and participants

This study, after registration and obtaining ethical approval (No. 51666-16471) from the Secretariat of the Biomedical Ethics Committee of Tabriz University, employed a convenience sampling method to recruit research participants from the visitors to Razi Hospital in Tabriz. The convenience sampling approach was chosen for its practicality and efficiency, ensuring the inclusion of participants who were readily available and willing to participate in the study. This method facilitated the recruitment of a sufficient number of participants within a reasonable timeframe.

The study population included paranoid schizophrenic patients, non-paranoid schizophrenic patients, their siblings (both paranoid and non-paranoid), and healthy controls. Initially, a total of 425 individuals were selected to assess eligibility. This process ensured that the required sample size was met after applying the inclusion and exclusion criteria.

Inclusion Criteria:

• Diagnosed with either paranoid or non-paranoid schizophrenia (for patient groups).
• Having a sibling diagnosed with schizophrenia (for sibling groups).
• No history of psychiatric disorders (for healthy control group).
• Aged between 20 and 60 years.

Exclusion Criteria:

• History of substance abuse.
• History of brain injury.
• Chronic physical ailments, excluding schizophrenia.

Participants were categorized based on their diagnosed sibling's schizophrenia subtype (paranoid or non-paranoid), ensuring that siblings themselves were not classified as paranoid or non-paranoid. Diagnosis of schizophrenia (paranoid and non-paranoid types) was based on DSM-IV criteria. The categorization of patients into paranoid and non-paranoid groups was based on clinical diagnostic interviews conducted by psychiatrists at Razi Hospital in Tabriz. All participants were recruited from the outpatient psychiatric department of Razi Hospital, and data were retrieved from their personal hospital profiles. According to their medical records, none of the patients had been hospitalized within the previous 6 months, and all had a diagnosis of schizophrenia for at least one year before the study. Symptom severity for all participants was recorded as moderate, and all patients were undergoing pharmacological treatment at the time of data collection. Furthermore, the participants' IQs were matched with an average of 80 to 90, based on the recorded hospital data and all participants had completed high school education (diploma level).

### 2.4 Ethical consideration

Participants were provided with detailed information about the study's goals, procedures, and potential benefits and risks, and were informed of their rights, including the ability to withdraw at any time without consequences. Informed consent was obtained, ensuring participants' voluntary agreement to participate. Confidentiality and anonymity were strictly maintained by using unique codes instead of personal identifiers, with data securely stored and accessible only to the research team. The study emphasized voluntary participation, reinforcing that participants could withdraw at any time without affecting their access to services. Moreover, participants received no financial incentive. Ethical principles, including respect for participants, beneficence, and justice, were upheld throughout the data collection process, ensuring the protection of participants' rights and well-being.

### 2.5 Material

The study employed the “Ekman 60 Faces Test” to evaluate participants' recognition of basic facial emotions, specifically focusing on negative emotions such as sadness, disgust, fear, surprise, and anger. The test is a well-established psychological tool, known for its validity in assessing emotional recognition and has demonstrated good test-retest reliability with a coefficient of 0.68 (Ekman and Friesen, 1976). The images used in the test are standardized, featuring actors displaying prototypical expressions, ensuring consistency across various studies and populations. The images used in the test have been validated across various cultural contexts, including populations in the Middle East, making them culturally appropriate for this study.

#### 2.5.1 Procedure for completing the test

During the test, each image was presented for 1 s, requiring participants to quickly and intuitively identify the emotion, mirroring real-world social interactions. The procedure involved displaying 60 images one by one in a distraction-free environment, with participants selecting the correct emotion from a list. Scores related to happiness and neutral expressions were excluded to maintain the focus on negative emotions. This decision was based on previous studies showing that negative emotions are more strongly associated with social dysfunction in schizophrenia. Participants' performance was scored based on the correct identification of the five negative emotions, with a maximum possible score of 50 points. To ensure participants understood the procedure, a brief training session was provided before the test, and no feedback was given during the test to avoid influencing responses.

### 2.6 Statistical analysis

The collected data were analyzed using descriptive statistics (mean and standard deviation) and inferential statistics, specifically the Multivariate Analysis of Variance (MANOVA). SPSS version 24 was used for all statistical analyses. The use of MANOVA was justified due to its ability to handle multiple dependent variables simultaneously, providing a more comprehensive understanding of the differences between the groups.

#### 2.6.1 Descriptive statistics

Descriptive statistics were computed to summarize the basic features of the data. These included the mean and standard deviation for each of the variables related to the ability to recognize negative facial expressions (sadness, fear, anger, and disgust) across the different groups (paranoid schizophrenic patients, non-paranoid schizophrenic patients, their siblings, and healthy controls).

#### 2.6.2 Assumptions testing

Before performing MANOVA, the assumptions of normality and homogeneity of variances were tested to ensure the validity of the analysis.

Normality: The Kolmogorov-Smirnov test was used to assess the normality of the distribution of each dependent variable. A non-significant result (*p* > 0.05) indicated that the data were normally distributed.

Homogeneity of Variances: Levene's test was conducted to evaluate the homogeneity of variances for each dependent variable across the groups. A non-significant result (p > 0.05) suggested that the variances were equal across the groups.

#### 2.6.3 MANOVA

MANOVA was employed to assess whether there are statistically significant differences in the dependent variables (ability to recognize sadness, fear, anger, and disgust) based on the independent variable (group: paranoid schizophrenic patients, non-paranoid schizophrenic patients, their siblings, and healthy controls). The MANOVA equation is given by:

The following equations were used in the MANOVA analysis:

Y=XB+E

where:

• Y is the matrix of dependent variables.

• X is the matrix of independent variables (groups).

• B is the matrix of coefficients (effects of the independent variable on the dependent variables).

• E is the matrix of errors (residuals).

The significance of the MANOVA was tested using Wilks' Lambda (Λ):

$$\Lambda =\frac{\mathrm{Det}\left(E\right)}{\mathrm{Det}\left(E+H\right)}$$

where:

• E is the matrix of errors.
• H is the matrix of the hypothesis.

#### 2.6.4 ANOVA

Following a significant MANOVA, one-way ANOVAs were conducted for each dependent variable to determine specific group differences. The equation used for the ANOVA is:

$$F=\frac{\mathrm{Ms}\;\mathrm{between}\;}{\mathrm{Ms}\;\mathrm{within}.}$$

where:

• F is the F-ratio.
• *MS* between is the mean square between groups.
• *MS* within is the mean square within groups.

#### 2.6.5 Post-hoc tests

Tukey post hoc tests were performed to make pairwise comparisons between group means, identifying where specific differences lie among the groups in their ability to recognize negative facial expressions.

This statistical approach provided a detailed analysis of the differences in recognizing negative facial expressions among paranoid schizophrenic patients, non-paranoid schizophrenic patients, their siblings, and healthy controls, contributing to a comprehensive understanding of social cognition deficits in schizophrenia.

## 3 Result

**Fig. 2:**
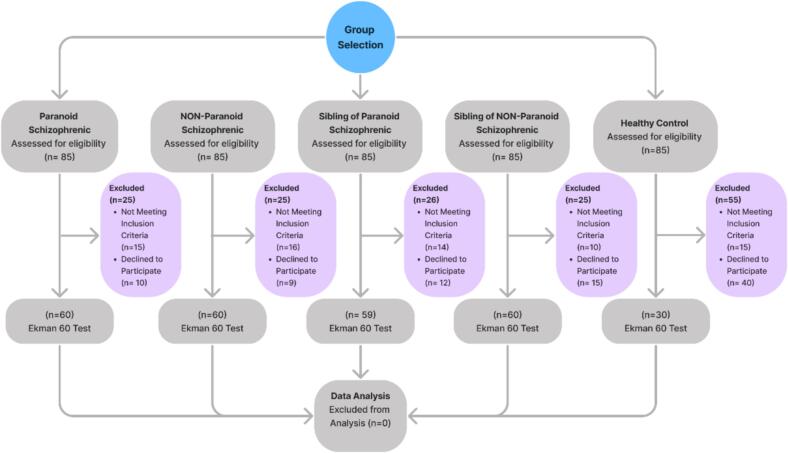
Consort Flow Diagram.

Fig. 2 illustrates that after categorizing participants into five groups (paranoid schizophrenic patients, non-paranoid schizophrenic patients, siblings of paranoid schizophrenic patients, siblings of non-paranoid schizophrenic patients, and healthy controls), 85 individuals in each group were selected for eligibility assessment.

In each group, an assessment process was conducted to determine the eligibility of participants. Participants who did not meet the inclusion criteria or declined to participate were excluded. In the group of paranoid schizophrenic patients, 25 individuals were excluded, with 15 not meeting the inclusion criteria and 10 declining to participate. In the group of non-paranoid schizophrenic patients, 25 individuals were excluded, with 16 not meeting the inclusion criteria and 9 declining to participate. In the group of siblings of paranoid schizophrenic patients, 26 individuals were excluded, with 14 not meeting the inclusion criteria and 12 declining to participate. In the group of siblings of non-paranoid schizophrenic patients, 25 individuals were excluded, with 10 not meeting the inclusion criteria and 15 declining to participate. In the healthy control group, 55 individuals were excluded, with 15 not meeting the inclusion criteria and 40 declining to participate.

After these exclusions, the final sample sizes for each group were as follows: 60 paranoid schizophrenic patients, 60 non-paranoid schizophrenic patients, 59 siblings of paranoid schizophrenic patients, 60 siblings of non-paranoid schizophrenic patients, and 30 healthy controls. All final participants completed the Ekman 60 Faces Test to assess their ability to recognize basic facial emotions. All participants who completed the Ekman 60 Faces Test were included in the data analysis phase, with no participants excluded from the analysis. This structured and systematic recruitment process ensured the inclusion of a representative sample for the comprehensive analysis of negative facial expression recognition abilities across different groups.

The table below reports the gender and age of participants categorized by the study groups.

Table 1 summarizes the demographic characteristics of the participants, including gender and age distribution. The chi-square test results indicate that there were no significant differences between the groups in terms of gender and age (p > 0.05), demonstrating homogeneity across these factors. This ensures that demographic differences did not confound the analysis of emotion recognition abilities.

**Table 1:** Demographic characteristics of study participants by group.

Demographic information	Healthy	Siblings of paranoid	Siblings of non-paranoid	Non-paranoid schizophrenia	Paranoid schizophrenia	χ^2^	Sig
Gender							
Female	16	32	34	23	25	6.26	0.182
Male	14	27	26	37	35		

Age							
20–30 years	7	15	16	13	12	1.84	0.989
31–40 years	9	19	17	20	19		
41–50 years	9	17	17	19	18		
51–60 years	5	8	10	8	11		

The results in Table 2 indicate that the paranoid and non-paranoid schizophrenia groups have lower mean scores in the components of the ability to recognize negative facial expressions compared to the siblings and healthy individuals.

**Table 2:** Mean and standard deviation of the components of the ability to recognize negative facial expressions by study group.

Group	Statistic	Ability to recognize sadness	Ability to recognize fear	Ability to recognize anger	Ability to recognize disgust	Ability to recognize negative states
Paranoid Schizophrenia	Mean	3.75	3.65	4.33	3.97	15.70
	Std Dev	1.02	1.69	1.43	1.51	3.39
Non-Paranoid Schizophrenia	Mean	4.40	3.97	4.40	3.67	16.43
	Std Dev	1.05	1.65	1.73	1.31	3.72
Siblings of non-paranoid	Mean	7.23	6.83	6.65	6.72	27.43
	Std Dev	0.91	1.53	1.36	1.98	3.60
Siblings of Paranoid	Mean	7.71	6.90	6.93	6.58	28.12
	Std Dev	0.79	1.51	1.32	1.82	3.14
Healthy	Mean	8.07	7.37	7.00	6.63	29.07
	Std Dev	0.83	1.63	2.03	2.50	5.64

The assumptions for ANOVA, including the normality of the dependent variable distribution and the homogeneity of variances, were examined. The results from the Kolmogorov-Smirnov test and the obtained significance levels for each research variable, which were >0.05, indicated that the distribution of the components of the ability to recognize negative facial expressions was normal. Additionally, the assumption of equal variances was met for all four subscales (p > 0.05).

Using multivariate analysis of variance (MANOVA), a significant effect of the group factor (independent variable) was found. This effect indicates that there is a difference in at least one of the components of the ability to recognize negative facial expressions among the four groups: schizophrenia, paranoid schizophrenia, siblings of schizophrenia patients, siblings of paranoid schizophrenia patients, and healthy individuals (Wilks' Lambda = 0.19, *p* < 0.01).

The results in Table 3 indicate that the differences among the four groups in the ability to recognize anger, fear, sadness, and disgust are confirmed. As shown in Table 3, the significance levels obtained for the ability to recognize anger, fear, sadness, and disgust are smaller than the significance level of 0.012 obtained from the Bonferroni correction (dividing the significance level of 0.05 by the 4 dependent variables). Subsequently, to compare the groups pairwise, the Tukey post hoc test was used.

**Table 3:** One-Way ANOVA tests within multivariate analysis of variance.

Variable	Source of Variation	SS	df	MS	F	Significance Level	Eta Squared
Ability to recognize sadness	Group	849.51	4	212.38	243.40	0.001	0.79
	Error	230.35	264	0.87			
Ability to recognize fear	Group	672.08	4	168.02	65.79	0.001	0.50
	Error	674.27	264	2.55			
Ability to recognize anger	Group	407.62	4	101.90	42.76	0.001	0.39
	Error	629.11	264	2.38			
Ability to recognize disgust	Group	534.74	4	133.68	42.07	0.001	0.39
	Error	838.82	264	3.18			

The results in Table 4 indicate that the differences between the non-paranoid and paranoid schizophrenia groups compared to other groups in the ability to recognize anger, fear, sadness, and disgust are significant (p < 0.05). Additionally, the difference between the paranoid schizophrenia and non-paranoid schizophrenia groups in recognizing sadness is significant (*p* < 0.05). However, the paranoid and non-paranoid schizophrenia groups do not show significant differences in recognizing anger, fear, and disgust (p > 0.05). There are no significant differences among the groups of siblings of non-paranoid, siblings of paranoid, and healthy individuals in the ability to recognize anger, fear, sadness, and disgust (*p* > 0.05).

**Table 4:** Pairwise comparison of group means in the components of the ability to recognize negative facial expressions.

Dependent variable	Group comparison	Mean difference	Standard error	Significance
Ability to Recognize Sadness	Paranoid Schizophrenia vs. Non-Paranoid Schizophrenia	−0.85	0.17	0.001
	Paranoid Schizophrenia vs. Siblings of Non-Paranoid	−3.48	0.17	0.001
	Paranoid Schizophrenia vs. Siblings of Paranoid	−3.96	0.17	0.001
	Paranoid Schizophrenia vs. Healthy	−4.32	0.21	0.001
	Non-Paranoid Schizophrenia vs. Siblings of Non-Paranoid	−2.83	0.17	0.001
	Non-Paranoid Schizophrenia vs. Siblings of Paranoid	−3.31	0.17	0.001
	Non-Paranoid Schizophrenia vs. Healthy	−3.67	0.21	0.001
	Siblings of Non-Paranoid vs. Siblings of Paranoid	−0.48	0.17	0.08
	Siblings of Non-Paranoid vs. Healthy	−0.53	0.21	0.06
	Siblings of Paranoid vs. Healthy	−0.36	0.21	0.44
Ability to Recognize Fear	Paranoid Schizophrenia vs. Non-Paranoid Schizophrenia	−0.32	0.29	0.81
	Paranoid Schizophrenia vs. Siblings of Non-Paranoid	−3.18	0.29	0.001
	Paranoid Schizophrenia vs. Siblings of Paranoid	−3.25	0.29	0.001
	Paranoid Schizophrenia vs. Healthy	−3.72	0.36	0.001
	Non-Paranoid Schizophrenia vs. Siblings of Non-Paranoid	−2.87	0.29	0.001
	Non-Paranoid Schizophrenia vs. Siblings of Paranoid	−2.93	0.29	0.001
	Non-Paranoid Schizophrenia vs. Healthy	−3.40	0.36	0.001
	Siblings of Non-Paranoid vs. Siblings of Paranoid	−0.07	0.29	0.98
	Siblings of Non-Paranoid vs. Healthy	−0.53	0.36	0.57
	Siblings of Paranoid vs. Healthy	−0.47	0.36	0.69
Ability to Recognize Anger	Paranoid Schizophrenia vs. Non-Paranoid Schizophrenia	−0.07	0.28	0.98
	Paranoid Schizophrenia vs. Siblings of Non-Paranoid	−2.32	0.28	0.001
	Paranoid Schizophrenia vs. Siblings of Paranoid	−2.60	0.28	0.001
	Paranoid Schizophrenia vs. Healthy	−2.67	0.35	0.001
	Non-Paranoid Schizophrenia vs. Siblings of Non-Paranoid	−2.25	0.28	0.001
	Non-Paranoid Schizophrenia vs. Siblings of Paranoid	−2.53	0.28	0.001
	Non-Paranoid Schizophrenia vs. Healthy	−2.60	0.35	0.001
	Siblings of Non-Paranoid vs. Siblings of Paranoid	−0.28	0.28	0.86
	Siblings of Non-Paranoid vs. Healthy	−0.35	0.35	0.85
	Siblings of Paranoid vs. Healthy	−0.07	0.35	0.98
Ability to Recognize Disgust	Paranoid Schizophrenia vs. Non-Paranoid Schizophrenia	0.30	0.33	0.89
	Paranoid Schizophrenia vs. Siblings of Non-Paranoid	−2.75	0.33	0.001
	Paranoid Schizophrenia vs. Siblings of Paranoid	−2.61	0.33	0.001
	Paranoid Schizophrenia vs. Healthy	−2.67	0.40	0.001
	Non-Paranoid Schizophrenia vs. Siblings of Non-Paranoid	−3.05	0.33	0.001
	Non-Paranoid Schizophrenia vs. Siblings of Paranoid	−2.91	0.33	0.001
	Non-Paranoid Schizophrenia vs. Healthy	−2.97	0.40	0.001
	Siblings of Non-Paranoid vs. Siblings of Paranoid	0.14	0.33	0.92
	Siblings of Non-Paranoid vs. Healthy	0.08	0.40	0.97
	Siblings of Paranoid vs. Healthy	−0.06	0.40	0.99

## 4 Discussion

This study aimed to investigate the recognition of negative facial expressions among different subgroups within the schizophrenia spectrum and their siblings. The results revealed significant impairments in emotion recognition among paranoid and non-paranoid schizophrenic patients compared to their siblings and healthy controls. Paranoid patients showed the most severe deficits, with a mean score of 3.75 in recognizing sadness, notably lower than the 8.07 observed in healthy controls. Non-paranoid patients scored slightly higher at 4.40. Siblings of paranoid and non-paranoid patients scored 7.71 and 7.23, respectively, which, while slightly lower than the mean score of the healthy controls, did not show a statistically significant difference. Similar trends were found across other emotions, including fear, anger, and disgust, where schizophrenic patients consistently scored lower than both their siblings and healthy controls.

The interpretation of these key findings suggests that the profound impairments in facial emotion recognition observed in schizophrenic patients highlight the impact of the disorder on social cognitive functions. Paranoid schizophrenic patients displayed more severe deficits in emotion recognition compared to non-paranoid patients, indicating subtype-specific cognitive impairments. However, contrary to our initial assumption, the findings do not support significant deficits in siblings relative to healthy controls. Therefore, the statement suggesting a genetic predisposition based on sibling performance has been revised. The ability to recognize negative emotions is crucial for effective social interactions, and these deficits likely contribute to social isolation and relationship difficulties in schizophrenic individuals.

The comparison with previous studies underscores the unique contributions of this research. Earlier studies have consistently documented deficits in facial emotion recognition among individuals with schizophrenia, particularly in recognizing negative emotions such as fear and anger (Fett et al., 2013; Fusar-Poli et al., 2022; Kohler et al., 2010). However, few studies have directly compared paranoid and non-paranoid subtypes within the schizophrenia spectrum, which this research addresses by examining the differential performance of these subgroups and including their siblings in the analysis. Our findings align with those of Fusar-Poli et al. (2022), who reported that subgroups within the schizophrenia spectrum may have distinct cognitive profiles, particularly in social cognition. These results support the idea that facial emotion recognition can serve as an intermediate phenotype for psychosis, reflecting both genetic vulnerability and the clinical manifestations of schizophrenia.

In contrast to previous research that broadly examined facial emotion recognition (Gao et al., 2021; Montag et al., 2007), this study emphasizes the specific challenges faced by individuals with different subtypes of schizophrenia. While studies such as Kohler et al. (2010) have established that individuals with schizophrenia generally struggle more with recognizing negative emotions, our findings show that these difficulties may be more pronounced in specific subgroups, particularly the non-paranoid subtype. This supports the hypothesis that distinct cognitive and emotional processing profiles exist within the schizophrenia spectrum (Fett et al., 2013), which has significant implications for developing tailored interventions.

Additionally, this study builds on previous research by exploring the familial and genetic contributions to social cognition deficits in schizophrenia. By including siblings of patients, we add to the literature on endophenotypes—measurable traits that link genetic risk with clinical outcomes (Flint and Munafò, 2007; Gottesman and Gould, 2003). Studies like Lavoie et al. (2013) and Fusar-Poli et al. (2022) have demonstrated that first-degree relatives of schizophrenia patients often exhibit social cognition impairments, though not as severe as those seen in patients themselves. The relatively intact abilities of siblings in this study further suggest that early interventions for at-risk individuals could mitigate potential social cognitive deficits and reduce the impact of genetic and environmental factors.

This research also contributes to the growing understanding of symptom formation theories in schizophrenia. The deficits in facial emotion recognition observed in our study, particularly in negative emotions, align with symptom formation models that suggest such impairments exacerbate psychotic symptoms and contribute to social dysfunction (Fett et al., 2013; Kohler et al., 2010). Recognizing these impairments as part of the broader symptomatology of schizophrenia can inform targeted therapeutic strategies aimed at improving social functioning and quality of life for patients.

Overall, this study provides a more nuanced understanding of the social cognitive deficits in schizophrenia by differentiating between subtypes and incorporating familial factors. These findings highlight the importance of developing personalized interventions based on the distinct cognitive and emotional profiles of schizophrenia subgroups, paving the way for future research into the heritable aspects of social cognition in this population.

Moreover, including siblings in this study sheds light on the genetic predisposition to social cognitive deficits and suggests that interventions aimed at improving emotional recognition could be beneficial even before clinical symptoms fully manifest. By focusing on early and preventive measures, particularly in those with a familial risk of schizophrenia, it may be possible to improve long-term outcomes and enhance resilience against environmental stressors.

These findings contribute to the growing body of literature on social cognition in schizophrenia by offering a dual perspective: while acknowledging the deficits, they also highlight the potential for intervention and improvement. This balanced approach underscores the need for a comprehensive understanding that not only identifies challenges but also seeks practical solutions to enhance the lives of those affected by schizophrenia. Future research should continue to explore both the limitations and the strengths within this population, aiming to develop holistic treatment approaches that address social cognitive deficits while fostering individual strengths.

Overall, the findings highlight the profound social cognitive impairments in schizophrenia, particularly in recognizing negative emotions, and suggest a genetic predisposition moderated by environmental factors. These insights contribute to the growing body of literature on social cognition in schizophrenia and provide a foundation for future research and interventions aimed at improving the social functioning of affected individuals.

### 4.1 Limitations

First, the use of convenience sampling at Razi Hospital in Tabriz, Iran, may introduce selection bias, limiting the generalizability of the findings to the broader population of individuals with schizophrenia and their relatives. Future studies should consider using random sampling techniques to enhance generalizability. Second, the study was conducted in a specific cultural and geographical context, which may influence the results. Cultural differences in facial emotion recognition and expression could affect the generalizability of the findings to other populations. Comparative studies across different cultures are needed to validate these results globally. Finally, the study's cross-sectional design limits the ability to draw causal inferences. Longitudinal studies are needed to understand the developmental trajectory of facial emotion recognition abilities in individuals with schizophrenia and their relatives over time.

## 5 Conclusion

This study addresses a critical gap in schizophrenia research by investigating the ability to recognize negative facial expressions among paranoid and non-paranoid schizophrenic patients, their siblings, and healthy controls. The innovation of this study lies in its comparative analysis across different subgroups within the schizophrenia spectrum and their first-degree relatives, providing valuable insights into the potential trait-related and genetic underpinnings of social cognition deficits.

The key findings reveal significant deficits in recognizing negative facial emotions among schizophrenic patients compared to siblings and healthy controls. Paranoid patients scored the lowest, with a mean of 3.75 for sadness, compared to 8.07 for healthy controls, and non-paranoid patients scored 4.40. Siblings of paranoid and non-paranoid patients had scores of 7.71 and 7.23, showing milder deficits. For fear, paranoid and non-paranoid patients scored 3.65 and 3.97, siblings scored 6.83 and 6.90, and healthy controls scored 7.37. In recognizing anger, patients scored around 4.33 to 4.40, siblings 6.65 to 6.72, and healthy controls 7.00. Lastly, for disgust, patients scored 3.97 and 3.67, while siblings scored 6.58 to 6.93, and controls 6.63.

Overall, the study demonstrates that schizophrenic patients exhibit significant impairments in recognizing negative facial emotions compared to their siblings and healthy controls. These findings suggest that the ability to recognize negative emotions is profoundly impaired in schizophrenic patients, contributing to their social cognitive deficits and social interaction challenges. These findings reinforce the need for targeted interventions that address the specific emotional recognition challenges faced by individuals with schizophrenia, especially those with paranoid symptoms. By focusing on these social cognitive impairments, therapeutic strategies can improve social functioning and overall quality of life for this population. Additionally, early identification and intervention in at-risk individuals, particularly siblings, could potentially mitigate the progression of these deficits.

## Funding/financial disclosure

This research did not receive any specific grant from funding agencies in the public, commercial, or not-for-profit sectors.

## CRediT authorship contribution statement

**Leila Shateri:** Project administration, Methodology, Investigation, Conceptualization. **Hamid Yari Renani:** Writing – review & editing, Methodology, Formal analysis, Data curation. **Abbas Bakhshipour Rudsari:** Supervision, Software. **Touraj Hashemi Nosratabad:** Visualization, Validation. **Zahra Saeidi:** Writing – original draft.

## Declaration of competing interest

The authors declare that there are no conflicts of interest regarding the publication of this paper. This research received no specific grant from funding agencies in the public, commercial, or not-for-profit sectors. All authors have contributed significantly to the work, have read and approved the final manuscript, and agree with its submission to the Schizophrenia Research Cognition Journal. The study's design, collection, analysis, interpretation of data, writing of the report, and the decision to submit the article for publication were conducted independently and not influenced by any external organization. No financial or other relationships could lead to a conflict of interest, including consultancies, employment, expert testimony, honoraria, patents (actual or pending), and royalties. The integrity of the research process and findings are upheld to the highest standards, ensuring objectivity, transparency, and reproducibility.

## Data Availability

The data used in this study are available from the corresponding author upon reasonable request.
